# Importance of ERK1/2 in Regulation of Protein Translation during Oocyte Meiosis

**DOI:** 10.3390/ijms19030698

**Published:** 2018-03-01

**Authors:** Jaroslav Kalous, Anna Tetkova, Michal Kubelka, Andrej Susor

**Affiliations:** 1 Institute of Animal Physiology and Genetics, Academy of Sciences of the Czech Republic, Rumburska 89, 27721 Libechov, Czech Republic; Tetkova@iapg.cas.cz (A.T.); Kubelka@iapg.cas.cz (M.K.); Susor@iapg.cas.cz (A.S.); 2 Department of Cell Biology, Faculty of Science, Charles University in Prague, Albertov 6, 12843 Prague 2, Czech Republic

**Keywords:** ERK1/2, MAP kinase, mTOR, translation, oocyte, eIF4E, CPEB1

## Abstract

Although the involvement of the extracellular signal-regulated kinases 1 and 2 (ERK1/2) pathway in the regulation of cytostatic factor (CSF) activity; as well as in microtubules organization during meiotic maturation of oocytes; has already been described in detail; rather less attention has been paid to the role of ERK1/2 in the regulation of mRNA translation. However; important data on the role of ERK1/2 in translation during oocyte meiosis have been documented. This review focuses on recent findings regarding the regulation of translation and the role of ERK1/2 in this process in the meiotic cycle of mammalian oocytes. The specific role of ERK1/2 in the regulation of mammalian target of rapamycin (mTOR); eukaryotic translation initiation factor 4E (eIF4E) and cytoplasmic polyadenylation element binding protein 1 (CPEB1) activity is addressed along with additional focus on the other key players involved in protein translation.

## 1. Introduction

Animal female germ cells—oocytes—are used as a convenient research model in cellular and developmental biology. Maturation of vertebrate oocytes into haploid gametes relies on two consecutive meiotic divisions without intervening DNA replication. During the growth period, the oocytes in ovarian follicles are kept in the prophase of meiosis I and accumulate constituents such as organelles, RNAs and proteins. When oocytes reach their full size, they undergo striking changes in nuclear morphology due to large-scale chromatin condensation. Histone marks associated with active chromatin are replaced by repressive histone modifications in the nucleus of the fully grown oocyte and the nucleus becomes transcriptionally inactive [1]. In the absence of transcription, the completion of meiosis and early embryo development rely on maternally synthetized mRNAs [2,3]. Thus, meiotic maturation of oocytes is dependent on the translational activation of stored maternal mRNAs which are translated later during oocyte maturation [4,5]. RNAs accumulated in oocytes are highly stable but more than 90% of these mRNAs are degraded after the first embryo division [6,7].

**Figure 1:**
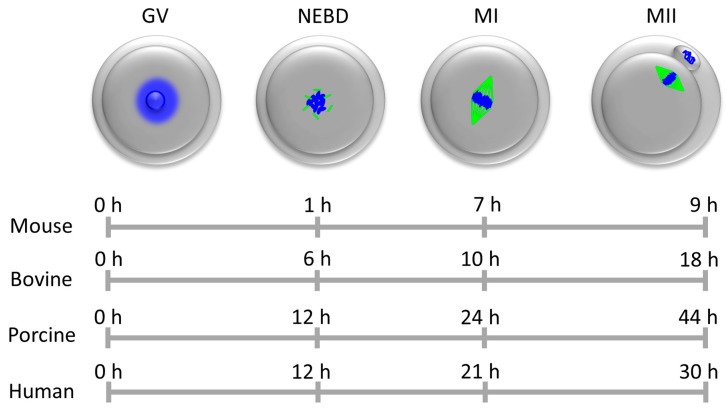
Time-sequence of meiotic maturation. The oocytes of most animal species are arrested in meiotic prophase I in so-called germinal vesicle (GV) stage, i.e., with an intact nuclear membrane. When oocytes begin meiotic maturation, the nuclear envelope breaks down (NEBD), the chromatin condenses and the first meiotic spindle forms (metaphase I, MI). Subsequently, oocytes pass through meiosis I, when the first polar body is extruded, and they are arrested in metaphase of meiosis II (MII) awaiting fertilization. Chromatin/chromosomes are depicted in blue, tubulin/spindle in green.

Thereafter specific stimuli cause the oocytes to resume meiosis and undergo meiotic maturation, before being arrested again during later stages of meiosis (in metaphase II) in preparation for fertilization (Figure 1) [8].

Meiotic maturation of oocytes involves a sequence of events—meiotic resumption, transitions of oocytes from G2 arrest to M-phase and then to meiosis II controlled by the interplay between cyclin-dependent kinases and extracellular signal-regulated protein kinases 1 and 2 (ERK1/2). When transcription is inhibited many maternal mRNAs are translationally activated during this process [9,10,11,12]. These temporally translated proteins play key roles in the principal meiotic events, such as reorganization of microtubules, meiotic spindle assembly, condensation and segregation of chromosomes, as well as meiosis II (MII) arrest [13,14]. The development of high-quality oocytes plays a pivotal role in determining the outcome of sexual reproduction. The quality of oocyte meiotic maturation is to a large extent dependent on protein synthesis as the regulation of gene expression at the transcription level at the meiosis stage is halted.

During oocyte maturation both the 7-methylguanylate (m^7^G) cap structure and the polyadenylated poly(A) tail are involved in repression and activation of protein translation. The m^7^G cap structure and the activities of its direct and indirect binding proteins, including the eIF4E binding protein 1 (4E-BP1), contribute to the regulation of maternal mRNA translation in the mouse oocyte. The m^7^G cap is present on the majority (80%) of mRNA molecules from unfertilized mouse oocytes and activities of m^7^G cap binding proteins are implicated in the regulation of maternal mRNA translation [15]. It has been postulated that translation of maternal mRNAs depends on the dynamic modulation of the poly(A) tail located at the 3′ end of the mRNA where the polyadenylation of dormant mRNAs induces translation [16,17].

## 2. MAP Kinases

**Figure 2:**
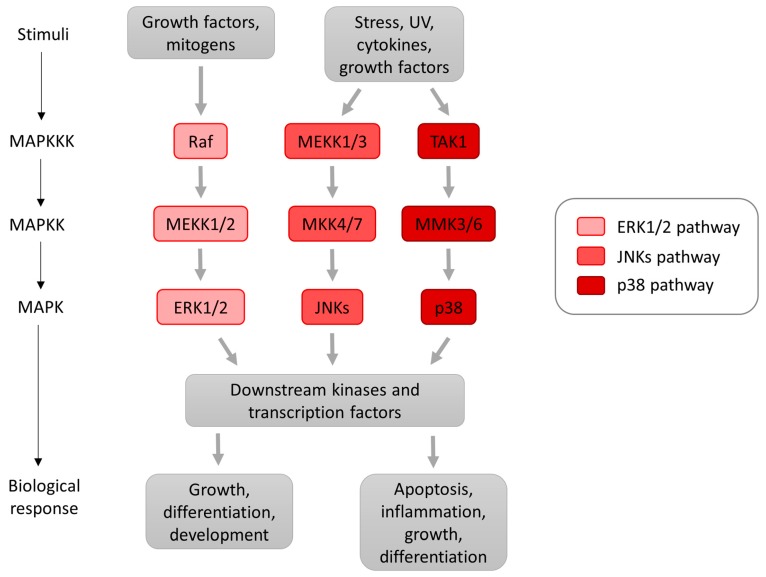
Schematic diagram of main mammalian mitogen-activated protein (MAP) kinase signalling pathways. Extracellular stimuli activate the MAP kinase pathways through mechanisms mediated by GTPases. Once MAP kinase kinase kinases (MAPKKKs), such as RAF proto-oncogene serine/threonine-protein kinase (Raf), mitogen-activated protein kinase kinase kinase 1 (MEKK1/3) and TAK (transforming growth factor β (TGFβ)-activated kinase) are activated, they phosphorylate MAP kinase kinases (MAPKKs) which then phosphorylate and activate the extracellular signal-regulated kinases 1 and 2 (ERK1/2), c-JUN N-terminal kinases (JNKs) and p38 kinases. Activated MAP kinases can translocate to the nucleus to phosphorylate a number of transcription factors. The ERK1/2 pathway is predominantly activated by growth factors, whereas stress and inflammatory cytokines preferentially activate the JNKs and p38 pathways. Arrows indicate direct stimulatory modification. UV: ultraviolet.

Mitogen-activated protein (MAP) kinases, evolutionarily conserved Ser/Thr kinases ubiquitously expressed in eukaryotes [18,19] regulate important cellular processes such as gene expression, proliferation, metabolism, apoptosis and immune defence [20,21]. The MAP kinase cascade is activated by consecutive phosphorylations—i.e., when stimulated, each MAP kinase is phosphorylated by an upstream MAP kinase. A MAP kinase cascade comprises of a MAP kinase (MAPKKK) activating a MAP kinases kinase (MAPKK) which then activates a MAP kinase (Figure 2) [18,22]. Phosphorylation of MAP kinase can be blocked by MAP kinase protein phosphatases (MKPs), which can dephosphorylate both the phosphotyrosine and phosphothreonine residues on MAP kinases [20,23].

The three most extensively studied MAP kinases in mammalian cells are the p38 MAP kinase α, β, γ, and δ isoforms; the c-JUN N-terminal kinase 1, 2 and 3 (JNKs); and the ERK1/2 [24,25,26].

Activation of p38 MAP kinase isoforms is induced by chemical and physical stimuli such as oxidative stress and ultraviolet (UV) irradiation (Figure 2). p38 MAP kinase is also activated by cytokines and in some cases, also by mitogen-activated protein kinase kinase 4 (MKK4), a kinase that is described as an activator of JNK [23]. Activated p38 MAP kinases can translocate from the cytosol to the nucleus where they phosphorylate the serine/threonine residues of the many substrates implicated in stress responses, growth inhibition and apoptosis [26]).

JNKs, also referred to as stress-activated kinases (SAPKs), were initially characterized by their activation in response to cell stressors such as UV irradiation. JNKs are involved in proliferation, differentiation, apoptosis and inflammation, and when dysregulated contribute to many diseases involving neurodegeneration, chronic inflammation, birth defects, cancer and ischemia/reperfusion injury [27].

ERK1/2 activation is initiated by the binding of a ligand to a receptor tyrosine kinase (RTK) at the cell plasma membrane followed by the activation of the small G-protein Rat sarcoma virus oncogene (Ras). Subsequently, Ras recruits and activates the RAF proto-oncogene serine/threonine-protein kinase (Raf), a MAPKKK, which activates the MEK (MAPKK), that then phosphorylates ERK1/2 on both threonine and tyrosine residues [28,29]. The Ras/Raf/MEK/ERK1/2 pathway can be deactivated by dual-specificity MAP kinase phosphatases [20,26].

ERK1 and ERK2 kinases (also called MAPK3 and MAPK1) are functionally redundant as they share all known substrates [30]. ERK1/2 phosphorylates and activates downstream kinases and other substrates, which influences the regulation of translation. This includes p90 ribosomal S6 kinase (RSK) and MAPK-interacting protein kinases MNK1 and MNK2 (MNKs) [31,32,33]. It has been shown that RSK and MNKs are implicated in the regulation of mRNA translation [34,35] as they phosphorylate and regulate a number of downstream targets—components of the translational machinery [33,36]. In human cells, RSK (downstream of the ERK1/2 pathway) and p70 S6 kinase (S6K; downstream of the phosphatidylinositol 3-kinase (PI3K)/protein kinase B (PKB)/mammalian target of rapamycin (mTOR) pathway phosphorylate the eukaryotic translation initiation factor 4B (eIF4B) at Ser422 [37,38]. Activated eIF4B enhances the activity of eukaryotic initiation complex 4F (eIF4F) by stimulating the eukaryotic translation initiation factor 4A (eIF4A), a RNA helicase that unwinds secondary structures on the 5′ untranslated region (5′ UTR) [39].

## 3. Role of MAPK in Regulation of Meiosis

**Figure 3:**
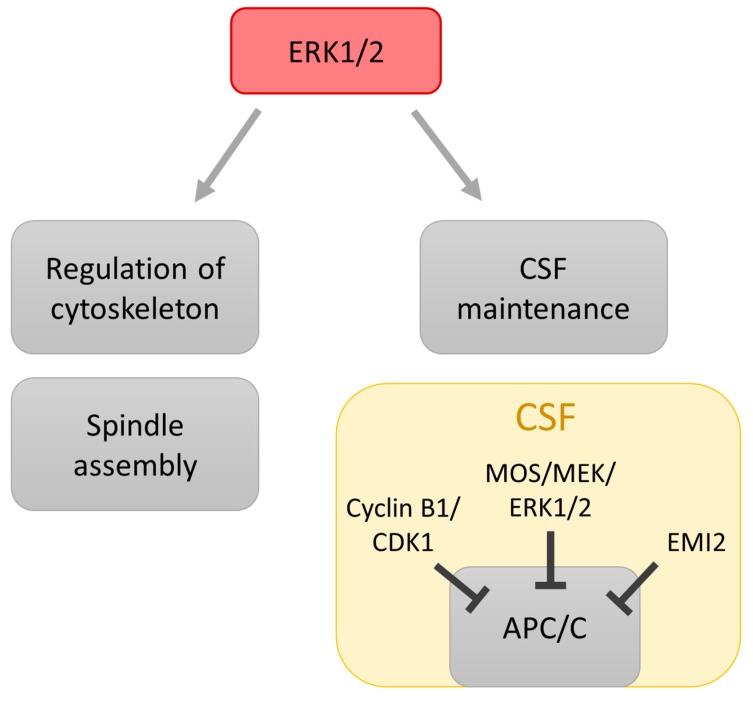
A role of the ERK1/2 in regulation of meiosis. During meiotic maturation ERK1/2 activity is essential for microtubule organization and meiotic spindle assembly (spindle depicted in green, chromosomes in blue). ERK1/2 is also an important component of the so-called cytostatic factor (CSF) protein complex, which prevents the exit of the oocytes from metaphase II stage via prevention of cyclin B destruction by anaphase-promoting complex/cyclosome (APC/C) complex. Moreover, downstream ERK1/2 effector p90 ribosomal S6 kinase (RSK) participates in maintaining the CSF arrest since RSK is involved in anaphase-promoting complex/cyclosome (APC/C) inhibition. Inhibitory modification of the kinases to APC/C is depicted as a blunt end line.

In the past years an intense effort has been made to elucidate the roles of the ERK1/2 cascade in the regulation of meiosis progression in oocytes but the identities of the ERK1/2 substrates in mammalian oocytes are yet to be fully revealed. In *Xenopus* oocytes ERK1/2 appears to be indispensable for the regulation of meiotic progress [40]. Although the involvement of ERK1/2 in the resumption of meiosis in mouse and porcine oocytes has not yet been confirmed [41,42], experiments with ERK1/2 inhibition and mouse oocyte knockouts produced severe impairment of microtubule organization and meiotic spindle assembly [43,44] (Figure 3).

When ERK1/2 or their upstream kinase MOS (Oocyte Maturation Factor Mos) were deleted, a precocious separation of sister chromatids and parthenogenetic activation of mouse oocytes occurred [45,46]. Efforts have been made to identify the ERK1/2 phosphorylation substrates present in the regulation of these processes. ERK1/2 substrates MISS (MAPK Interacting and Spindle Stabilizing) and DOC1R (Deleted in Oral Cancer 1 Related) have been identified as regulating microtubule organization in mouse oocytes in metaphase MII (MII oocytes) [47,48]. There is clear evidence that ERK1/2 is a part of the so-called cytostatic factor (CSF), a protein complex which prevents the exit of oocytes from the metaphase II stage via the stabilization of cyclin B (Figure 3) [45,46]. CSF is responsible for the establishment and persistence of the metaphase of MII (MII arrest) of unfertilized vertebrate oocytes. CSF activity occurs during meiosis II and ceases after fertilization [49]. It was found that MOS is required for CSF activity in oocytes [50]. The oocytes from MOS-deficient mice did not arrest at MII but instead underwent spontaneous parthenogenetic activation and abortive development [51]. Moreover, ERK1/2 did not become activated during the maturation of such oocytes and maturation promoting factor (MPF) activity declined prematurely at MII [52]. CSF arrest at MII is mediated by a sole ERK1/2 target, the protein kinase RSK [53,54]. Inhibition of the anaphase-promoting complex/cyclosome (APC/C) is required in order to maintain CSF arrest. APC/C is an M-phase E3 ubiquitin ligase that targets M-phase B type cyclins and securin (regulator of sister chromatid cohesion) for degradation at the metaphase/anaphase transition [55]. During MII arrest, APC activation is blocked by the prevention of the binding of the APC activator Cdc20/Fizzy protein [56,57]. Inhibition of Cdc20/Fizzy binding to APC is mediated by products of the *Bub* and *Mad* genes [58]. RSK phosphorylates and activates the budding uninhibited by benzimidazoles (BUB1) protein kinase, which may cause metaphase arrest due to the inhibition of the APC/C by a direct binding of BUB1 to Cdc20/Fizzy, a conserved mechanism defined genetically in yeast and mammalian cells. CSF arrest in vertebrate oocytes induced by RSK provides a link between the ERK1/2 pathway and the spindle assembly checkpoint in the meiotic cell cycle [59,60]. It has been evidenced that ERK1/2 is important player in cytoplasmic maturation of oocytes. Impairment of cytoplasmic maturation in sheep oocytes was revealed if ERK1/2 activity was inhibited during in vitro maturation resulting in a subsequent decrease of cleavage rate and blastocyst development [61]. The level of ERK1/2 activity plays an important role during aging of MII-oocytes. When the in vitro culture of porcine oocytes was prolonged up to 72 h to induce aging of oocytes a significant decrease of the ERK1/2 activity occurred during the first 12 h of aging with following decrease during prolonged culture. It has been suggested that a premature decrease of ERK1/2 activity in aged MII porcine oocytes negatively influenced subsequent early embryo development as proportions of oocytes with abnormal anaphase II were significantly increased after parthenogenic activation of aged oocytes [62,63].

The function of ERK1/2 during meiotic maturation of mammalian oocytes, which has been up-to-now described, include: regulation of microtubules and spindle assembly [45,64], stabilization of MPF [65,66] involvement in so-called cytostatic which is responsible for the block of oocytes in MII prior fertilization by the sperm [45,46,47,48,49,50,51,52,53,54,55,56,57,58,59,60,61,62,63,64,65,66,67].

## 4. Translational Regulation at 5′ End of mRNA (Cap-Dependent Initiation of Protein Translation)

**Figure 4:**
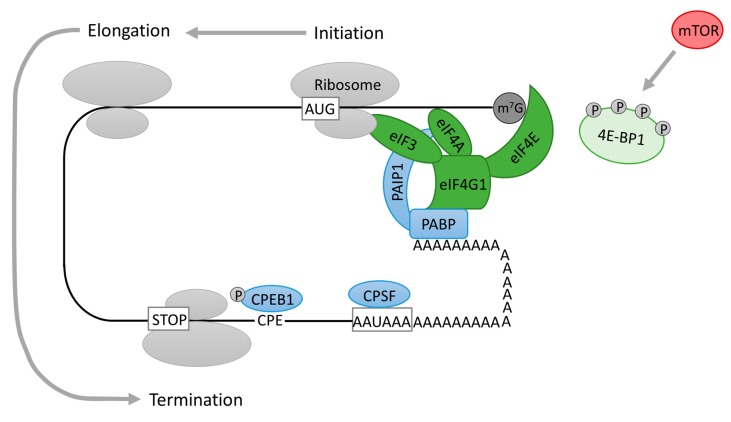
Scheme of a polyadenylation-induced translation. The cytoplasmic polyadenylation element (CPE) attaches to the cytoplasmic polyadenylation element binding protein 1 (CPEB1). Activated CPEB1 stabilizes the binding of activated cytoplasmic polyadenylation specific factor (CPSF) to the hexanucleotide sequence. Subsequently, CPSF recruits poly(A) polymerase (PAP) to the end of mRNA, where PAP catalyses poly(A) addition. eIF4E binding protein 1 (4E-BP1), when hyperphosphorylated by mammalian target of rapamycin (mTOR), dissociates from eukaryotic translation initiation factor 4E (eIF4E) and the formation of the eukaryotic translation initiation complex 4F (eIF4F) complex is enabled. Elongated poly(A) tail associates with a cytoplasmic poly(A) binding protein (PABP), which in turn stabilizes the cap binding eIF4F consisting of the mRNA cap-binding protein eIF4E, scaffolding protein eIF4G1 and RNA helicase (eIF4A). PABP binds poly(A) binding protein interacting protein 1 (PAIP1), which can bind eIF4A and the ribosome recruiting eukaryotic translation initiation factor 3 (eIF3). The closed loop complex enhances translation by increasing eIF4A and eIF3 recruitment. This leads to enhanced translation mediated by the poly(A) tail. Factors associated with cap-dependent translation initiation are depicted in green, poly(A) tail factors in blue, and kinases in red.

The initiation of protein translation is likely to be the most important step in the regulation of translation and a number of eukaryotic initiation factors (eIFs) are involved in this process. Transcribed eukaryotic messenger RNAs (mRNAs) have the 7-methylguanylate (m^7^G) cap structure at the 5′ end and are posttranscriptionally modified by the addition of ~250 adenine residues to form the poly(A) tail at the 3′ end [68]. Both m^7^G cap structure and poly(A) tail are implicated in the concerted repression and activation of protein translation [69,70] (Figure 4). Additionally, the interaction of factors bound to the m^7^G cap and poly(A) tail mediates mRNA circularization enhancing the efficiency of protein translation [17]. Protein interaction with both 5′ and 3′ ends of mRNA and mRNA circularization will be discussed further with regards to protein translation in oocytes and the involvement of the ERK1/2 pathway in these events.

When mRNA is transported from the nucleus to the cytoplasm the 5′ m^7^G cap structure is recognised by eIF4F, a trimeric protein complex composed of eukaryotic translation initiation factor 4E (eIF4E), which binds to the m^7^G cap; eIF4A, a helicase necessary for unwinding the secondary structure of mRNA; and a bridging protein eukaryotic initiation factor 4G1 (eIF4G1) [69,71] (Figure 4). eIF4G1, the largest component of eIF4F, is responsible for the integrity of the eIF4F complex, as well as providing binding sites for eIF3, MNKs and the poly(A)-binding protein [36,72]. eIF4B supports eIF4F activity by enhancing eIF4A RNA helicase activity [71,73]. eIF4G1 also bridges the mRNA with the ribosome through its interaction with the eukaryotic translation initiation factor 3 (eIF3) [74,75], which has been observed to interact directly with eIF4B [76,77]. The availability of eIF4E for binding the mRNA cap structure is regulated by eIF4E binding proteins (4E-BPs), which, in their hypophosphorylated state, inhibit protein translation by binding to eIF4E and preventing its association with cap and eIF4G1 [78]. The most studied 4E-BP is 4E-BP1 which, when hyperphosphorylated, dissociates from eIF4E and the formation of the eIF4F complex is enabled, resulting in stimulation of cap-dependent translation initiation. 4E-BP1 activity is regulated by the mTOR dependent phosphorylation [79] (Figure 4). At low mTOR activity, 4E-BP1 is hypophosphorylated and binds to eIF4E preventing the initiation of translation. When mTOR is activated, 4E-BP1 becomes phosphorylated on several serine and threonine amino acid residues and the release of 4E-BP1 from eIF4E enables the commencement of cap-dependent translation. A maximal hyperphosphorylation of 4E-BP1 is necessary however, in order to disrupt the association with eIF4E [80]. Also, mTOR can directly activate S6K which then activates a downstream target, ribosomal protein S6 (RPS6), leading to the initiation of protein synthesis [81]. Apart from the main role of mTOR in the positive regulation of protein translation by 4E-BP1, the above-mentioned implication of mTOR in eIF4B phosphorylation in somatic cells reveals more complex mTOR involvement. However, the role of eIF4B in translation during meiotic maturation of oocytes is yet to be fully described.

Another level of regulation of cap-dependent translation by phosphorylation is the phosphorylation of eIF4E itself. It has been assumed that phosphorylation of eIF4E at Ser209 stabilizes the binding of eIF4E to the m^7^G cap structure [82,83], however, it has been also revealed that phosphorylation of eIF4E reduces its affinity for the m^7^G cap [84,85]. eIF4E phosphorylation may be required for the release of eIF4F from the cap complex during the initiation process. Alternatively, phosphorylation of eIF4E may be involved in reprogramming translation by releasing the eIF4F complex to promote the chance of less abundant mRNAs to bind to ribosomes [84,86,87].

Strictly eIF4E-dependent mRNAs, so-called eIF4E sensitive transcripts, often possess long, highly structured 5′ UTR or 5′-terminal oligopyrimidine (TOP) tracts [88] and encode mostly regulatory proteins. Ref [89] show that mTOR almost entirely regulates the translation of transcripts with established TOP tracts. The cap-dependent translation is allowed to occur when 4E-BP1, hyperphosphorylated by mTOR is released from eIF4E. Hence it is not surprising that both the increase and decrease of global mRNA translation correlates with mTOR activity [80,81,82,83,84,85,86,87,88,89,90,91].

## 5. Translational Regulation at the 3′ End of mRNA

Poly(A) tail elongation following the export of an mRNA to the cytoplasm is called cytoplasmic polyadenylation and was first discovered in frog oocytes and embryos. Polyadenylation of the 3′ UTR in the cytoplasm is largely correlated with mRNA stability and translational activation of mRNA. The stored mRNAs have short poly(A) tails, which must be elongated by poly(A) polymerase (PAP) before translation. Cytoplasmic polyadenylation requires the hexanucleotide polyadenylation signal (AAUAAA) and a U-rich cytoplasmic polyadenylation element (CPE). In maturing *Xenopus* oocytes CPE hexanucleotid UUUUA(A)U is implicated in translation [92,93]. The CPE is attached to the cytoplasmic polyadenylation element binding protein 1 (CPEB1) [94,95]) (Figure 4). When phosphorylated and activated, CPEB1 stabilizes the binding of activated cytoplasmic polyadenylation specific factor (CPSF) to the hexanucleotide sequence [96,97]. Subsequently, CPSF attracts PAP to catalyse poly(A) elongation. The polyadenylation of mRNA finally results in the initiation of protein translation at the mRNA 5′ cap.

When mRNA is transported from the nucleus to the cytoplasm the m^7^G cap structure is specifically recognized by the eIF4E (and as such with eIF4F) and the poly(A) tail at the 3′ end of mRNA binds to cytoplasmic poly(A)-binding proteins (PABPs). Both eIF4E and PABPs interact also with eIF4G1 creating a protein bridge between the two transcript ends (Figure 4). The pseudo-circularized structure of mRNA enhances the affinity of eIF4E to the cap and hence translation is initiated (Figure 4). 3′ UTR polyadenylation is essential for the regulation of translation initiation as circularization of mRNA is only possible when the mRNA is polyadenylated [16,17]. Circularization of mRNA promotes translation by enhancing the affinity of eIF4E to the m^7^G cap and also by enabling the ribosome to be used again when a translation run is completed [98].

## 6. eIF4E Activity in Oocytes

**Figure 5:**
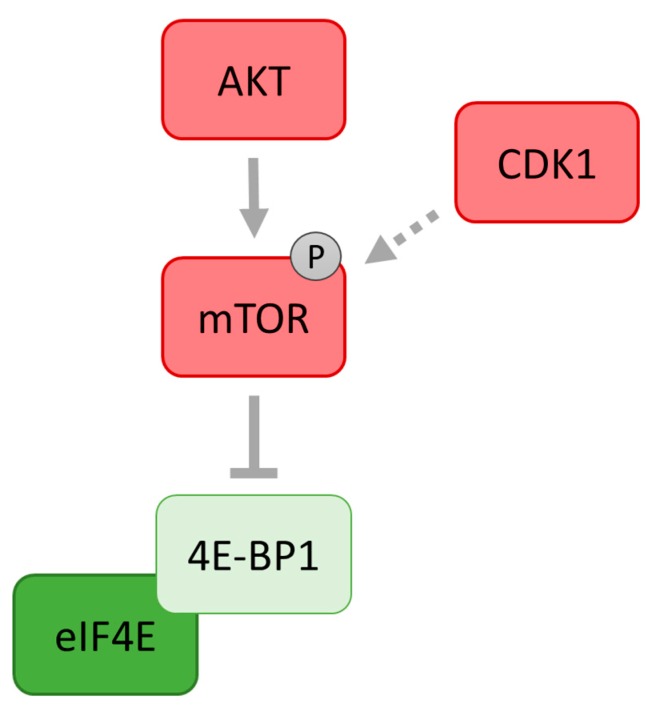
Cyclin-dependent kinase 1 (CDK1) participates at mTOR activation. In mitotic cells, CDK1 phosphorylates the regulatory associated protein of mTOR (RAPTOR), the key mTOR binding partner. In oocytes, CDK1 phosphorylates mTOR at Ser2448. This reinforces proposals that mTOR activity is highly regulated by CDK1 activity during cell cycle progression. Stimulatory modification is depicted as an arrow, inhibitory modification as a blunt end line. Dashed line with arrow indicates tentative stimulatory modification.

As documented in somatic cells, hypophoshorylated eIF4E binding proteins (4E-BPs) sequester eIF4E and prevent its association with eIF4G1 [78]. Similarly, 4E-BP1 reduces its affinity to the m^7^G cap structure [99]. It has been shown that 4E-BP1 becomes phosphorylated during the meiotic maturation of pig, bovine and mouse oocytes [100,101,102,103]. 4E-BP1 at the level of protein is the only member of the 4E-BP family present in maturing mouse oocytes [103]. The main effector kinases of 4E-BP1 phosphorylation/inactivation are mTOR and cyclin-dependent kinase 1 (CDK1) (Figure 5) which become highly active after the resumption of meiosis in mouse, human and bovine oocytes [103,104,105]. 4E-BP1 is phosphorylated by mTOR kinase and CDK1 suggesting that these two kinases stimulate cap-dependent translation during the course of meiosis [99,102,103]. It has been found that polo-like kinase 1 (PLK1) partially regulates 4E-BP1 phosphorylation at the MI and MII spindles in mouse oocytes and inhibition of PLK1 activity leads to the disruption of normal spindle formation and function [106]. The activity of mTOR is decreased at the cessation of meiosis after fertilization which is followed by the activation of 4E-BP1 as a repressor of cap-dependent translation [102]. Similarly, experiments with starfish oocytes revealed that dissociation of the eIF4E/4E-BP complex is transitory and the heterodimeric complex is restored before the first polar body emission and remains unchanged during the completion of meiosis [107]. This reinforces the theory that mTOR activity is highly regulated by cell cycle progression. In mouse oocytes disruption of mTOR/eIF4F signalling does not affect the progress of oocyte meiosis to the MII stage, although defects in spindle morphology and chromosome alignment were observed, suggesting that the synthesis of specific proteins is required for proper spindle formation and correct distribution of chromosomes during meiosis I [102].

## 7. Role of ERK1/2 in eIF4E Activation in Meiotic Cells

MNKs, downstream effectors of ERK1/2, regulate in somatic cells the assembly of eIF4F on the m^7^G cap and mRNA translation through direct phosphorylation of eIF4E [33,108,109]. Here, in response to both stress and proliferation signals, MNK1 and MNK2 directly phosphorylate eIF4E at the single site (Ser209 residue) localized at the carboxyl terminus [33,109,110]. Small interfering RNA mediated knockdown of protein phosphatase 2A (PP2A) or pharmacological inhibition of PP2A result in an increased phosphorylation of its target MNK1 and subsequently elevated phosphorylation of eIF4E [111]. The eIF4E phosphorylation of Ser209 residue is considerably enhanced when MNKs are bound to eIF4G1 [72,112].

**Figure 6:**
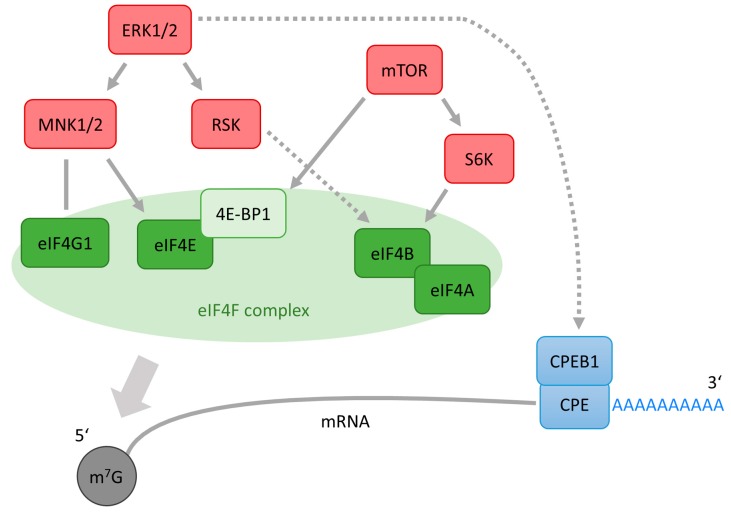
ERK1/2 and mTOR regulate protein translation in oocytes. MAPK-interacting kinase (MNK1), a downstream target of ERK1/2 stabilizes the binding of eIF4E to the m7G cap by phosphorylation eIF4E at the Ser209 residue. The eIF4E phosphorylation on Ser209 is considerably enhanced when MNK1 and MNK2 (MNKs) are bound to eIF4G1. Although downstream members of both ERK1/2 and mTOR pathways phosphorylate eIF4B in somatic cells, involvement of ERK1/2 in eIF4B phosphorylation was not reported in oocytes. In *Xenopus* oocytes ERK1/2 phosphorylates CPEB1 at four residues but not on Ser174, a key residue for activation of CPEB1. In mouse, a role of ERK1/2 in CPEB1 phosphorylation was confirmed only in cumulus-enclosed oocytes. Factors associated with cap-dependent translation initiation are depicted in green, poly(A) tail factors in blue, and kinases in red. Stimulatory modification is depicted as an arrow, inhibitory modification as a blunt end line. Dashed line with arrow indicates tentative stimulatory modification.

A similar mechanism was also found to operate in mammalian oocytes during meiotic maturation. In bovine oocytes eIF4E phosphorylation is under the control of ERK1/2 and closely correlates with ERK1/2 activation [101]. Also, in porcine oocytes MNK1 activated by the ERK1/2 signalling cascade has been shown to directly phosphorylate eIF4E [113] (Figure 6). Here, phosphorylation of eIF4E occurs approximately at the same time or just prior to metaphase of MI and tightly correlates with the activation of both MNK1 and ERK1/2 (but not p38 MAP kinase) [113].

Furthermore, inhibition of either mTOR or MNKs activity reduces protein synthesis in pachytene spermatocytes but not in round spermatids suggesting that the mTOR and MNK pathways regulate eIF4F assembly in meiotic male germ cells [110]. These results indicate that in these cells, mRNA translation is differentially dependent on the mTOR and MNK pathways in meiotic and post-meiotic male germ cells.

## 8. Cytoplasmic Polyadenylation of mRNA in Oocytes

The polyadenylation of the poly(A) tail occurs during the main events that control mRNA translation in vertebrate germ cells and early embryos [114]. Cytoplasmic polyadenylation regulates the translation of maternal mRNAs containing a cytoplasmic polyadenylation element (CPE) that binds specific trans-acting proteins [9]. CPEs are present at the 3′ UTR of many maternal mRNAs and they are implicated in the regulation of poly(A) tail length. CPEB1 belongs to the key oocyte factors that regulate maternal mRNA translation during oocyte maturation. In *Xenopus* and mouse oocytes CPEB1 mediates cytoplasmic polyadenylation of many CPE-containing mRNAs [115]. Although CPEB1 and the target maternal mRNAs are present in germinal vesicle (GV) stage oocytes (G2 phase), the majority of these mRNAs are polyadenylated and translated into proteins only after the resumption of meiosis [4,116]. However, it has been documented that CPEB1 is involved in resumption of meiosis and cyclin B translation in porcine oocytes [117].

In *Xenopus* oocytes CPE in complex with CPEB1 promotes either repression or activation of protein translation. CPEB1 inhibits translation by preventing the assembly of the m^7^G cap complex and by recruiting proteins that either compete for cap binding with eIF4E or compete with eIF4G1 for an interaction with eIF4E (e.g., 4E-BP1) [118,119]. Cytoplasmic polyadenylation regulates the translation of proteins essential for meiotic divisions, such as MOS and cyclins [4,120]. After meiotic resumption phosphorylation of CPEB1 on several serine/threonine residues is essential for early activation of many maternal mRNAs. In mouse oocytes activation of CPEB1 triggers the translation of many CPE-containing key maternal mRNAs including those encoding B-cell translocation gene 4 (*BTG4*), microtubule nucleation factor (*TPX2*) and deleted in azoospermia-like (*DAZL*), that are essential for oocyte maturation and maternal-zygotic transition [3,121,122]. However, a large fraction (70–90%) of CPEB1 proteins undergo a polyubiquitination-dependent degradation during meiosis, resulting in a changed CPEB/CPE ratio and hence enabling the activation of another class of mRNAs [96,123].

## 9. A Role of ERK1/2 in mRNA Polyadenylation in Oocytes

ERK1/2 plays a substantial role in the polyadenylation of mRNA in oocytes. In *Xenopus* oocytes ERK1/2 phosphorylates CPEB1 in four residues (Thr22, Thr164, Ser184, Ser248) but probably not on Ser174, a key residue for the activation of CPEB1 function [96,124]. When *Xenopus* oocytes are activated by progesterone, the kinase Aurora A phosphorylates the Ser174 residue of CPEB1 and increases the affinity of CPEB1 for cleavage [70]. However, results obtained on porcine oocytes are contradictory as it has been suggested that Aurora A kinase is either involved [117] or not involved in CPEB1 phosphorylation in this mammalian species [125].

In mouse oocytes, it has been shown that ERK1/2-triggered phosphorylation of Ser181 and Ser207 residues is essential for the onset of CPEB1 phosphorylation and protein translation during meiotic progression (Figure 6). Insufficient translation of maternal mRNAs including *Dazl*, *Tpx2* and *Btg4* is the main reason for the developmental defects detected in ERK1/2-inhibited mouse oocytes [126]. In sea urchin oocytes inhibition of ERK1/2 activity correlated with an inhibition of global protein synthesis and it has been suggested that ERK1/2 activity is required for synthesis of protein(s) implicated in chromatin/microtubule attachment [127]. On the other hand, it has also been revealed that cytoplasmic polyadenylation of cyclin B1 mRNA precedes ERK1/2 activation in maturing cumulus-free mouse oocytes, indicating that ERK1/2 activity is not likely to be required for the activation of cytoplasmic polyadenylation [128]. Experimental inhibition of ERK1/2 activity in mouse cumulus-free oocytes did not affect cytoplasmic polyadenylation nor translation of cyclin B1 mRNA. The data obtained on porcine oocytes did not confirm nor exclude the possible role of ERK1/2 in CPEB1-mediated cytoplasmic polyadenylation (Figure 6) [128]. However, when ERK1/2 was inhibited in cumulus-enclosed mouse oocytes, a substantial reduction of cyclin B1 mRNA poly(A) tail length was observed [128]. This finding is in accordance with the conclusion that cumulus cells surrounding the mouse oocyte are implicated in the regulation of maternal mRNAs translation [11]. In particular, the accumulation of TPX2 protein during the meiotic maturation of mouse cumulus-free oocytes is markedly reduced compared to that of cumulus-enclosed oocytes [11]. The results of [128] are in contrast with the data presented by [126] who proposed that ERK1/2 inhibition impairs cyclin B1 mRNA polyadenylation in maturing mouse oocytes. However, it is not clear if [126] carried out the experiments on in vitro cultured cumulus-free or cumulus-enclosed mouse oocytes.

The possible involvement of ERK1/2 in the regulation of RNA-binding protein DAZL has also been addressed. DAZL was identified as a CPEB1 downstream translational activator in mouse oocytes [129]. Mutations in DAZL expressed in prenatal and postnatal mouse male and female germ cells result in infertility and sterility [130,131]. TPX2 induces microtubule nucleation and acts as an indispensable regulator of centrosome and spindle pole assembly [132,133]. In ERK1/2-deficient oocytes the accumulation of TPX2 is impaired as its protein translation requires DAZL [126].

## 10. Cross-Talk between ERK1/2 and PI3K/mTOR Pathways

**Figure 7:**
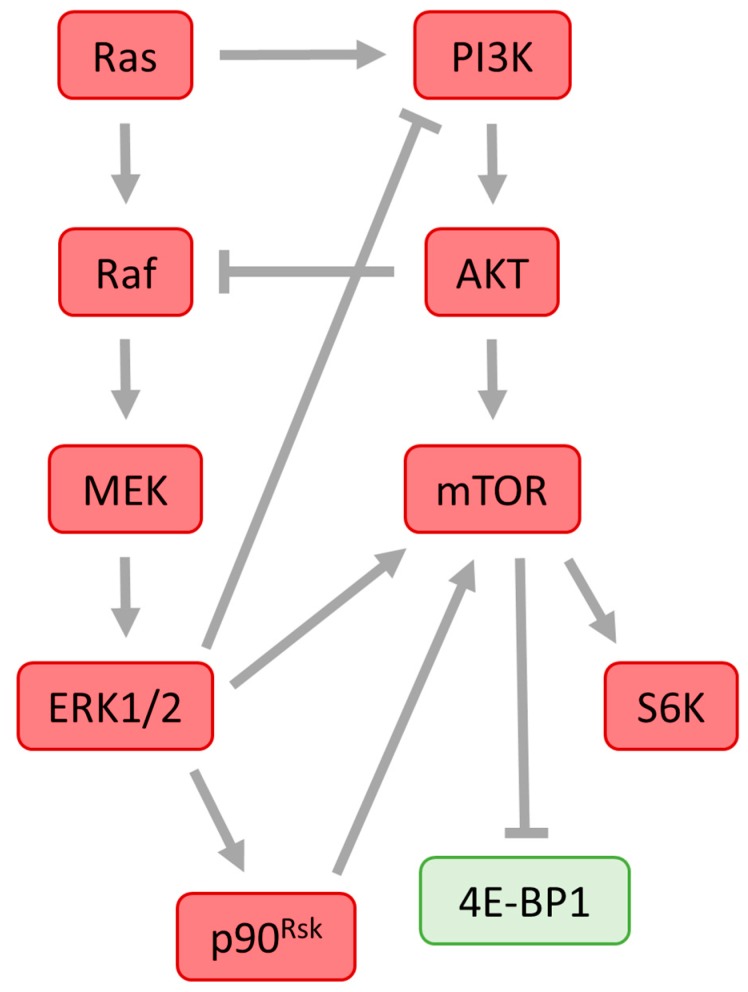
Crosstalk of the Ras/ERK1/2 and PI3K/mTOR pathways. Ras can directly bind and activate PI3K. Protein kinase AKT negatively regulates ERK1/2 activation by phosphorylating inhibitory sites in the Raf N-terminus. ERK1/2 negatively attenuates growth factor-induced AKT activation probably by GAB1-mediated recruitment of PI3K to the growth factor receptor. The Ras/ERK1/2 pathway is also able to cross-activate PI3K/mTOR by regulating PI3K and mTOR. Intensive activation of the Ras-ERK1/2 pathway stimulates mTOR activity. Positive regulation of the substrate protein is shown as an arrow and negative regulation of the substrate protein is depicted as a blunt end line.

The Raf/MEK/ERK1/2 and PI3K/PKB/mTOR pathways mediate cell survival, proliferation, metabolism and motility [134,135]. PKB, also known as AKT, is a serine/threonine-specific protein kinase activated by PI3K. PKB controls many downstream substrates including mTOR [136]. PI3K/PKB/mTOR and Raf/MEK/ERK1/2 pathways can act on the same substrate in a concerted manner [137] (Figure 7).

Both Raf/MEK/ERK1/2 and PI3K/PKB/mTOR pathways can be activated by G-protein coupled receptors or through receptor tyrosine kinase. Cross-talk between both pathways can occur at the receptor level [133,134]. Compensatory activation of PI3K and ERK1/2 signalling pathways has been documented indicating that PI3K/PKB/mTOR and Raf/MEK/ERK1/2 signalling pathways are not independent but interactive [135].

The Ras/Raf/MEK/ERK1/2 pathway cross-activates PI3K/PKB/mTOR signalling by regulating PI3K and mTOR at several points (see also Figure 7). Ras can directly bind and allosterically activate PI3K [136]. Intensive activation of the Ras-ERK1/2 pathway can also stimulate mTOR activity by ERK1/2 and RSK signalling to the tuberous sclerosis complex 2 (TSC2), which is sensitive to different growth factors and stress signals [137]. ERK1/2 and RSK can also stimulate the regulatory associated protein of mTOR (RAPTOR), a component of mTOR signalling which promotes the phosphorylation of 4E-BP1 by mTOR [138,139].

Also, scaffolding proteins of the members of the ERK1/2 cascade can regulate the mTOR-signalling pathway at several levels. MEK scaffolding protein 1 (MP1), which scaffolds MEK and ERK1/2, can support co-localization of ERK1/2 and mTOR pathway components and promote cross-talk between these two pathways [137,140]. The kinase suppressor of Ras (KSR) acts as a scaffold protein and co-localizes with Raf, MEK and ERK1/2 during ERK1/2 activation [140]. It has been documented that KSR also interacts with mTOR, RAPTOR and the TSC2-activating kinases AMPK and GSK3 [141,142].

The ERK1/2 and PI3K/PKB/mTOR pathways can negatively regulate each other’s activity. Both Ras/Raf/ERK1/2 and PI3K/PKB/mTOR pathways possess mechanisms that can negatively feed onto the other [137] (Figure 7). ERK1/2 negatively attenuates growth factor-induced PKB activation probably by GAB1-mediated recruitment of PI3K to the growth factor receptor [138]. The Ras/ERK1/2 pathway is also able to cross-activate PI3K/mTOR by regulating PI3K and mTOR. Ras can directly bind and activate PI3K [139]. In contrast, PKB negatively regulates ERK1/2 activation by phosphorylating inhibitory sites in the Raf N-terminus [140,141]. This inhibitory phosphorylation of Raf is blocked during mitogen-stimulated Raf activation [142].

The above-mentioned data suggest that cross-activation, cross inhibition and convergence of both Raf/MEKK/ERK1/2 and PI3K/PKB/mTOR pathways probably also play important roles in the regulation of protein translation.

It has been shown that CDK1 directly phosphorylates the key mTOR binding partner RAPTOR during mitosis and meiosis [103]. This reinforces proposals that mTOR activity is highly regulated by cell cycle progression.

ERK1/2, which is also able to regulate mTOR [138,139] also becomes active after the resumption of meiosis and remains so throughout meiotic maturation [143]. Thus, it appears that both the ERK1/2 and the PI3K/PKB/mTOR pathways converge to modulate translation of maternal mRNAs.

## 11. Perspectives

Translational regulation promotes the fine-tuning of processes in specialized cells, especially in cells without active transcription. Although valuable insights into the mechanism of regulation of translation has come from *Xenopus* oocytes, this understanding has only been extrapolated to the mammalian system. In order to address the dissimilarities and overlaps in transcriptome and proteome composition which might contribute to the molecular physiology of oocytes from different organisms, further studies are required. Comparative studies in mammalian model systems will provide important information regarding the components and mechanisms that may play critical regulatory roles in the physiology and pathology at specific cell stages. Consequent extrapolation of the findings from model mammalian oocytes to human oocytes might be beneficial for clinical applications.

In the future, it will be interesting to identify in the mammalian oocyte a subset of transcripts which translation is positively regulated through the ERK1/2 and mTOR axis. Accumulation of translationally controlled cell cycle regulators is rapid because the transcription step has already occurred. For many key cell cycle regulators and spindle assembly components, translational control represents an additional mechanism to precisely adjust their abundance. It remains to be discovered how all these different steps of translational control are integrated to temporally produce specific proteins essential for the meiotic progression of the oocyte.

Another important challenge in the research of the molecular physiology of the oocytes will be the genome-wide analysis of transcripts which have been translated in the oocytes from aged females. Reproductive aging is characterized by a marked decline in oocyte quality that contributes to infertility, miscarriages and birth defects. Surprisingly, ref. [144] show age-associated changes of an increased number of ribosomes in the oocytes from older females. Ribosome assembly is the process tightly connected with the initiation of translation and polyadenylation where ERK1/2 and the PI3K/PKB/mTOR pathways play the key role. Thus, this could have implications for the influence on oocyte quality.

Another exciting question can be addressed using the recently developed tools for detection in in situ translation [145]. The oocyte, as a one of the largest cells in the body, might also utilize spatial translational control which might contribute to the modulation of local events in spindle assembly or promote asymmetric division in the meiotic division/s. The specific localization and thus function of the key cap-dependent translation regulatory factors [102,146,147] is essential for the translation of specific mRNAs at the spindle area to ensure errorless cell cycle progression.

Answers to the many open questions regarding the interplay between translational regulation and meiotic progression will ultimately make a major contribution to our understanding of the molecular machinery involved in the two meiotic divisions and are essential in order to elucidate the basis of genetic errors.

## Abbreviations

ERK1/2	The extracellular regulated kinases 1 and 2
MAPK	Mitogen activated protein kinase
MAPKK	MAP kinases kinase
MAPKKK	MAP kinase
RSK	p90 ribosomal S6 kinase
MNKs	MAPK-interacting kinases MNK1 and MNK2
S6K	p70 S6 kinase
CSF	Cytostatic factor
mTOR	mammalian target of rapamycin
eIFs	Eukaryotic initiation factors
eIF4F	Eukaryotic translation initiation complex 4F
eIF4E	Eukaryotic translation initiation factor 4E
eIF4B	Eukaryotic translation initiation factor 4B
eIF4A	Eukaryotic translation initiation factor 4A
eIF4G1	Eukaryotic initiation factor 4G1
4E-BPs	EIF4E-binding proteins
4E-BP1	EIF4E
CPE	Cytoplasmic polyadenylation element
CPEB	CPE-binding protein
CPEB1	CPE-binding protein 1
PI3K	Phosphatidylinositol 3-kinase
PKB	Protein kinase B
MOS	Oocyte maturation factor Mos
MISS	MAPK interacting and spindle stabilizing
DOC1R	Deleted in oral cancer 1 related
APC/C	Anaphase-promoting complex/cyclosome
MPF	Maturation promoting factor
m7G	7-methylguanylate
MI	Meiosis I
MII	Meiosis II
PAP	Poly(A) polymerase
CPSF	Cytoplasmic polyadenylation specific factor
PP2A	Protein phosphatase 2A
Ras	Rat sarcoma virus oncogene
Raf	MEK kinase Raf
KSR	Kinase suppressor of Ras
MP1	MEK scaffolding protein 1
TSC 2	Tuberous sclerosis complex 2
RAPTOR	Regulatory associated protein of mTOR

## References

[1] Tanaka M., Kihara M., Hennebold J.D., Eppig J.J., Viveiros M.M., Emery B.R., Carrell D.T., Kirkman N.J., Meczekalski B., Zhou J. (2005). H1FOO is coupled to the initiation of oocytic growth. Biol. Reprod..

[2] Alizadeh Z., Kageyama S.I., Aoki F. (2005). Degradation of maternal mRNA in mouse embryos: Selective degradation of specific mRNAs after fertilization. Mol. Reprod. Dev..

[3] Chen J., Melton C., Suh N., Oh J.S., Horner K., Xie F., Sette C., Blelloch R., Conti M. (2011). Genome-wide analysis of translation reveals a critical role for deleted in azoospermia-like (Dazl) at the oocyte-to-zygote transition. Genes Dev..

[4] Sheets M.D., Fox C.A., Hunt T., Vande Woude G., Wickens M. (1994). The 3′-untranslated regions of c-mos and cyclin mRNAs stimulate translation by regulating cytoplasmic polyadenylation. Genes Dev..

[5] Richter J.D., Lasko P. (2011). Translational control in oocyte development. Cold Spring Harb. Perspect. Biol..

[6] Clarke H.J. (2012). Mouse Development.

[7] Hamatani T., Daikoku T., Wang H., Matsumoto H., Carter M.G., Ko M.S.H., Dey S.K. (2004). Global gene expression analysis identifies molecular pathways distinguishing blastocyst dormancy and activation. Proc. Natl. Acad. Sci. USA.

[8] Mehlmann L.M. (2005). Stops and starts in mammalian oocytes: Recent advances in understanding the regulation of meiotic arrest and oocyte maturation. Reproduction.

[9] Piqué M., López J.M., Foissac S., Guigó R., Méndez R. (2008). A Combinatorial Code for CPE-Mediated Translational Control. Cell.

[10] Gosden R., Lee B. (2010). Portrait of an oocyte: Our obscure origin. J. Clin. Investig..

[11] Chen J., Torcia S., Xie F., Lin C.J., Cakmak H., Franciosi F., Horner K., Onodera C., Song J.S., Cedars M.I. (2013). Somatic cells regulate maternal mRNA translation and developmental competence of mouse oocytes. Nat. Cell Biol..

[12] Piccioni F., Zappavigna V., Verrotti A.C. (2005). Translational regulation during oogenesis and early development: The cap-poly(A) tail relationship. C. R. Biol..

[13] Barkoff A.F., Dickson K.S., Gray N.K., Wickens M. (2000). Translational control of cyclin B1 mRNA during meiotic maturation: Coordinated repression and cytoplasmic polyadenylation. Dev. Biol..

[14] Gorbsky G.J. (2015). The spindle checkpoint and chromosome segregation in meiosis. FEBS J..

[15] Schultz G.A., Clough J.R., Johnson M.H. (1980). Presence of cap structures in the messenger RNA of mouse eggs. Development.

[16] Radford H.E., Meijer H.A., de Moor C.H. (2008). Translational control by cytoplasmic polyadenylation in Xenopus oocytes. Biochim. Biophys. Acta (BBA) Gene Regul. Mech..

[17] Brook M., Smith J.W.S., Gray N.K. (2009). The DAZL and PABP families: RNA-binding proteins with interrelated roles in translational control in oocytes. Reproduction.

[18] Kyriakis J.M., Avruch J. (2012). Mammalian MAPK Signal Transduction Pathways Activated by Stress and Inflammation: A 10-Year Update. Physiol. Rev..

[19] Peti W., Page R. (2013). Molecular basis of MAP kinase regulation. Protein Sci..

[20] Liu Y., Shepherd E.G., Nelin L.D. (2007). MAPK phosphatases—Regulating the immune response. Nat. Rev. Immunol..

[21] Arthur J.S.C., Ley S.C. (2013). Mitogen-activated protein kinases in innate immunity. Nat. Rev. Immunol..

[22] Johnson G.L. (2011). Defining MAPK interactomes. ACS Chem. Biol..

[23] Pimienta G., Pascual J. (2007). Canonical and alternative MAPK signalling. Cell Cycle.

[24] Chen Z., Cobb M.H. (2001). Regulation of stress-responsive mitogen-activated protein (MAP) kinase pathways by TAO2. J. Biol. Chem..

[25] Pearson G., Robinson F., Beers Gibson T., Xu B., Karandikar M., Berman K., Cobb M.H. (2001). Mitogen-Activated Protein (MAP) Kinase Pathways: Regulation and Physiological Functions. Endocr. Rev..

[26] Owens D.M., Keyse S.M. (2007). Differential regulation of MAP kinase signalling by dual-specificity protein phosphatases. Oncogene.

[27] Johnson G.L., Nakamura K. (2007). The c-jun kinase/stress-activated pathway: Regulation, function and role in human disease. Biochim. Biophys. Acta (BBA) Mol. Cell Res..

[28] Shaul Y.D., Seger R. (2007). The MEK/ERK cascade: From signalling specificity to diverse functions. Biochim. Biophys. Acta (BBA) Mol. Cell Res..

[29] Knight T., Irving J.A.E. (2014). Ras/Raf/MEK/ERK Pathway Activation in Childhood Acute Lymphoblastic Leukemia and Its Therapeutic Targeting. Front. Oncol..

[30] Roskoski R. (2012). ERK1/2 MAP kinases: Structure, function, and regulation. Pharmacol. Res..

[31] Gaestel M. (2016). MAPK-Activated Protein Kinases (MKs): Novel Insights and Challenges. Front. Cell Dev. Biol..

[32] Anjum R., Blenis J. (2008). The RSK family of kinases: Emerging roles in cellular signalling. Nat. Rev. Mol. Cell Biol..

[33] Waskiewicz A.J., Flynn A., Proud C.G., Cooper J.A. (1997). Mitogen-activated protein kinases activate the serine/threonine kinaseses Mnk1 and Mnk2. EMBO J..

[34] Roux P.P., Topisirovic I. (2012). Regulation of mRNA translation by signalling pathways. Cold Spring Harb. Perspect. Biol..

[35] Buxade M., Morrice N., Krebs D.L., Proud C.G. (2008). The PSF·p54nrb complex is a novel Mnk substrate that binds the mRNA for tumor necrosis factor α. J. Biol. Chem..

[36] Scheper G.C., Morrice N.A., Kleijn M., Proud C.G. (2001). The Mitogen-Activated Protein Kinase Signal-Integrating Kinase Mnk2 Is a Eukaryotic Initiation Factor 4E Kinase with High Levels of Basal Activity in Mammalian Cells The Mitogen-Activated Protein Kinase Signal-Integrating Kinase Mnk2 Is a Eukaryotic Initi. Mol. Cell. Biol..

[37] Andreou A.Z., Harms U., Klostermeier D. (2017). eIF4B stimulates eIF4A ATPase and unwinding activities by direct interaction through its 7-repeats region. RNA Biol..

[38] Magnuson B., Ekim B., Fingar D.C. (2012). Regulation and function of ribosomal protein S6 kinase (S6K) within mTOR signalling networks. Biochem. J..

[39] Sonenberg N., Hinnebusch A.G. (2013). Regulation of Translation Inition in Eukaryotes: Mechanisms and Biological Targrts. Cell.

[40] Gotoh Y., Masuyama N., Dell K., Shirakabe K., Nishida E. (1995). Initiation of Xenopus oocyte maturation by activation of the mitogen-activated protein kinase cascade. J Biol. Chem..

[41] Kubelka M., Anger M., Kalous J., Schultz R.M., Motlík J. (2002). Chromosome condensation in pig oocytes: Lack of a requirement for either cdc2 kinase or MAP kinase activity. Mol. Reprod. Dev..

[42] Kalous J., Kubelka M., Motlík J. (2003). The effect of PD98059 on MAPK regulation in cumulus-enclosed and cumulus-free mouse oocytes. Zygote.

[43] Fan H.-Y., Sun Q.-Y. (2004). Involvement of Mitogen-Activated Protein Kinase Cascade During Oocyte Maturation and Fertilization in Mammals1. Biol. Reprod..

[44] Zhang Y.L., Liu X.M., Ji S.Y., Sha Q.Q., Zhang J., Fan H.Y. (2015). ERK1/2 Activities Are Dispensable for Oocyte Growth but Are Required for Meiotic Maturation and Pronuclear Formation in Mouse. J. Genet. Genom..

[45] Verlhac M.H., Kubiak J.Z., Weber M., Géraud G., Colledge W.H., Evans M.J., Maro B. (1996). Mos is required for MAP kinase activation and is involved in microtubule organization during meiotic maturation in the mouse. Development.

[46] Paronetto M.P., Giorda E., Carsetti R., Rossi P., Geremia R., Sette C. (2004). Functional interaction between p90Rsk2 and Emi1 contributes to the metaphase arrest of mouse oocytes. EMBO J..

[47] Lefebvre C., Emilie Terret M., Djiane A., Rassinier P., Maro B., Verlhac M.H. (2002). Meiotic spindle stability depends on MAPK-interacting and spindle-stabilizing protein (MISS), a new MAPK substrate. J. Cell Biol..

[48] Terret M.E. (2003). DOC1R: A MAP kinase substrate that control microtubule organization of metaphase II mouse oocytes. Development.

[49] Schmidt A. (2006). Cytostatic factor: An activity that puts the cell cycle on hold. J. Cell Sci..

[50] Posada J., Yew N., Ahn N.G., Vande Woude G.F., Cooper J.A. (1993). Mos stimulates MAP kinase in Xenopus oocytes and activates a MAP kinase kinase in vitro. Mol. Cell. Biol..

[51] Hashimoto N., Watanabe N., Furuta Y., Tamemoto H., Sagata N., Yokoyama M., Okazaki K., Nagayoshi M., Takeda N., Ikawa Y. (1994). Parthenogenetic activation of c-mos mice. Nature.

[52] Choi T., Rulong S., Resau J., Fukasawa K., Matten W., Kuriyama R., Mansour S., Ahn N., Vande Woude G.F. (1996). Mos/mitogen-activated protein kinase can induce early meiotic phenotypes in the absence of maturation-promoting factor: A novel system for analyzing spindle formation during meiosis I. Proc. Natl. Acad. Sci. USA..

[53] Gross S.D., Lewellyn A.L., Maller J.L. (2001). A Constitutively Active Form of the Protein Kinase p90Rsk1 Is Sufficient to Trigger the G2/M Transition in Xenopus Oocytes. J. Biol. Chem..

[54] Bhatt R.R., Ferrell J.E. (2016). The Protein Kinase p90 Rsk as an Essential Mediator of Cytostatic Factor Activity. Science.

[55] Zachariae W., Nasmyth K. (1999). Whose end is destruction: Cell division and the anaphase- promoting complex. Genes Dev..

[56] Prinz S., Hwang E.S., Visintin R., Amon A. (1998). The regulation of Cdc20 proteolysis reveals a role for the APC components Cdc23 and Cdc27 during S phase and early mitosis. Curr. Biol..

[57] Fang G., Yu H., Kirschner M.W. (1998). The checkpoint protein MAD2 and the mitotic regulator CDC20 form a ternary complex with the anaphase-promoting complex to control anaphase initiation. Genes Dev..

[58] Vanoosthuyse V., Valsdottir R., Javerzat J.-P., Hardwick K.G. (2004). Kinetochore targeting of fission yeast Mad and Bub proteins is essential for spindle checkpoint function but not for all chromosome segregation roles of Bub1p. Mol. Cell. Biol..

[59] Maller J.L., Schwab M.S., Gross S.D., Taieb F.E., Roberts B.T., Tunquist B.J. (2002). The mechanism of CSF arrest in vertebrate oocytes. Mol. Cell. Endocrinol..

[60] Schwab M.S., Roberts B.T., Gross S.D., Tunquist B.J., Taieb F.E., Lewellyn A.L., Maller J.L. (2001). Bub1 is activated by the protein kinase p90(Rsk) during Xenopus oocyte maturation. Curr. Biol..

[61] Ni H., Sheng X., Cui X., Gu M., Liu Y., Qi X., Xing S., Guo Y. (2015). Epidermal growth factor-mediated mitogen-activated protein Kinase3/1 pathway is conducive to in vitro maturation of sheep oocytes. PLoS ONE.

[62] Ebeling S., Labudda A., Meinecke B. (2010). In vitro ageing of porcine oocytes: Changes in phosphorylation of the mitogen-activated protein kinase (MAPK) and parthenogenetic activability. Reprod. Domest. Anim..

[63] Ma W., Zhang D., Hou Y., Li Y.-H., Sun Q.-Y., Sun X.-F., Wang W.-H. (2005). Reduced expression of MAD2, BCL2, and MAP kinase activity in pig oocytes after in vitro aging are associated with defects in sister chromatid segregation during meiosis II and embryo fragmentation after activation. Biol. Reprod..

[64] Sun S.C., Xiong B., Lu S.S., Sun Q.Y. (2008). MEK1/2 is a critical regulator of microtubule assembly and spindle organization during rat oocyte meiotic maturation. Mol. Reprod. Dev..

[65] Miyagaki Y., Kanemori Y., Baba T. (2011). Possible involvement of mitogen- and stress-activated protein kinase 1, MSK1, in metaphase-II arrest through phosphorylation of EMI2 in mouse oocytes. Dev. Biol..

[66] Tiwari M., Gupta A., Sharma A., Prasad S., Pandey A.N., Yadav P.K., Pandey A.K., Shrivastav T.G., Chaube S.K. (2018). Role of Mitogen Activated Protein Kinase and Maturation Promoting Factor During the Achievement of Meiotic Competency in Mammalian Oocytes. J. Cell. Biochem..

[67] Suzuki T., Suzuki E., Yoshida N., Kubo A., Li H., Okuda E., Amanai M., Perry A.C.F. (2010). Mouse Emi2 as a distinctive regulatory hub in second meiotic metaphase. Development.

[68] Munroe D., Jacobson A. (1990). mRNA poly(A) tail, a 3′ enhancer of translational initiation. Mol. Cell. Biol..

[69] Richter J.D., Sonenberg N. (2005). Regulation of cap-dependent translation by eIF4E inhibitory proteins. Nature.

[70] Mendez R., Hake L.E., Andresson T., Littlepage L.E., Ruderman J.V., Richter J.D. (2000). Phosphorylation of CPE binding factor by Eg2 regulates translation of c- mos mRNA. Nature.

[71] Gingras A., Raught B., Sonenberg N. (1999). eIF4 Initiation Factors: Effectors of mRNA Recruitment to Ribosomes and Regulators. Annu. Rev. Biochem..

[72] Pyronnet S., Imataka H., Gingras A.C., Fukunaga R., Hunter T., Sonenberg N. (1999). Human eukaryotic translation initiation factor 4G (eIF4G) recruits Mnk1 to phosphorylate eIF4E. EMBO J..

[73] Harms U., Andreou A.Z., Gubaev A., Klostermeier D. (2014). EIF4B, eIF4G and RNA regulate eIF4A activity in translation initiation by modulating the eIF4A conformational cycle. Nucleic Acids Res..

[74] Etchison D., Milburn S.C., Edery I., Sonenberg N., Hershey J.W. (1982). Inhibition of HeLa cell protein synthesis following poliovirus infection correlates with the proteolysis of a 220,000-dalton polypeptide associated with eucaryotc initiation factor 3 and a cap binding protein complex. J. Biol. Chem..

[75] Pain V.M. (1996). Initiation of protein synthesis in eukaryotic cells. Eur. J. Biochem..

[76] Méthot N., Song M.S., Sonenberg N. (1996). A region rich in aspartic acid, arginine, tyrosine, and glycine (DRYG) mediates eukaryotic initiation factor 4B (eIF4B) self-association and interaction with eIF3. Mol. Cell. Biol..

[77] Vornlocher H., Hanachi P., Ribeiro S. (1999). A 110-Kilodalton Subunit of Translation Initiation Factor eIF3 and an Associated 135-kilodalton Protein Are Encoded by theSaccharomyces cerevisiae TIF32 and TIF31Genes. J. Biol. Chem..

[78] Pause A., Belsham G.J., Gingras A.-C., Donzé O., Lin T.-A., Lawrence J.C., Sonenberg N. (1994). Insulin-dependent stimulation of protein synthesis by phosphorylation of a regulator of 5′-cap function. Nature.

[79] Josse L., Xie J., Proud C.G., Smales C.M. (2016). mTORC1 signalling and eIF4E/4E-BP1 translation initiation factor stoichiometry influence recombinant protein productivity from GS-CHOK1 cells. Biochem. J..

[80] Gingras A.C., Raught B., Gygi S.P., Niedzwiecka A., Miron M., Burley S.K., Polakiewicz R.D., Wyslouch-Cieszynska A., Aebersold R., Sonenberg N. (2001). Hierarchical phosphorylation of the translation inhibitor 4E-BP1. Genes Dev..

[81] Vander Haar E., Lee S.I., Bandhakavi S., Griffin T.J., Kim D.H. (2007). Insulin signalling to mTOR mediated by the Akt/PKB substrate PRAS40. Nat. Cell Biol..

[82] Marcotrigiano J., Gingras A.C., Sonenberg N., Burley S.K. (1997). Cocrystal Structure of the Messenger RNA 5′ Cap-Binding Protein (eIF4E) Bound to 7-methyl-GDP. Cell.

[83] Matsuo H., Li H., McGuire A.M., Mark Fletcher C., Gingras A.C., Sonenberg N., Wagner G. (1997). Structure of translation factor elF4E bound to m7GDP and interaction with 4E-binding protein. Nat. Struct. Biol..

[84] Scheper G.C., Proud C.G. (2002). Does phosphorylation of the cap-binding protein eIF4E play a role in translation initiation?. Eur. J. Biochem..

[85] Slepenkov S.V., Darzynkiewicz E., Rhoads R.E. (2006). Stopped-flow kinetic analysis of eIF4E and phosphorylated eIF4E binding to cap analogs and capped oligoribonucleotides: Evidence for a one-step binding mechanism. J. Biol. Chem..

[86] Morley S.J., Naegele S. (2002). Phosphorylation of eukaryotic initiation factor (eIF) 4E is not required for de novo protein synthesis following recovery from hypertonic stress in human kidney cells. J. Biol. Chem..

[87] Zuberek J., Jemielity J., Jablonowska A., Stepinski J., Dadlez M., Stolarski R., Darzynkiewicz E. (2004). Influence of Electric Charge Variation at Residues 209 and 159 on the Interaction of eIF4E with the mRNA 5′ Terminus. Biochemistry.

[88] Dowling R., Topisirovic I., Alain T., Bidinosti M. (2010). mTORC1-mediated cell proliferation, but not cell growth, controlled by the 4E-BPs Supplemental data. Science.

[89] Thoreen C.C., Chantranupong L., Keys H.R., Wang T., Gray N.S., Sabatini D.M. (2012). A unifying model for mTORC1-mediated regulation of mRNA translation. Nature.

[90] Walsh D., Mohr I. (2014). Coupling 40S ribosome recruitment to modification of a cap-binding initiation factor by eIF3 subunit e. Genes Dev..

[91] Naegele S., Morley S.J. (2004). Molecular cross-talk between MEK1/2 and mTOR signalling during recovery of 293 cells from hypertonic stress. J. Biol. Chem..

[92] McGrew L.L., Dworkin-Rastl E., Dworkin M.B., Richter J.D. (1989). Poly(A) elongation during Xenopus oocyte maturation is required for translational recruitment and is mediated by a short sequence element. Genes Dev..

[93] Fox C.A., Sheets M.D., Wickens M.P. (1989). Poly(A) addition during maturation of frog oocytes: Distinct nuclear and cytoplasmic activities and regulation by the sequence UUUUUAU. Genes Dev..

[94] Paris J., Swenson K., Piwnica-Worms H., Richter J.D. (1991). Maturation-specific polyadenylation: In vitro activation by p34(cdc2) and phosphorylation of a 58-kD CPE-binding protein. Genes Dev..

[95] Hake L.E., Richter J.D. (1994). CPEB is a specificity factor that mediates cytoplasmic polyadenylation during Xenopus oocyte maturation. Cell.

[96] Mendez R., Barnard D., Richter J.D. (2002). Differential mRNA translation and meiotic progression require Cdc2-mediated CPEB destruction. EMBO J..

[97] Hodgman R., Tay J., Mendez R., Richter J.D. (2001). CPEB phosphorylation and cytoplasmic polyadenylation are catalyzed by the kinase IAK1/Eg2 in maturing mouse oocytes. Development.

[98] Tomek W., Wollenhaupt K. (2012). The “closed loop model” in controlling mRNA translation during development. Anim. Reprod. Sci..

[99] Siemer C., Smiljakovic T., Bhojwani M., Leiding C., Kanitz W., Kubelka M., Tomek W. (2009). Analysis of mRNA associated factors during bovine oocyte maturation and early embryonic development. Mol. Reprod. Dev..

[100] Ellederova Z., Kovarova H., Melo-Sterza F., Livingstone M., Tomek W., Kubelka M. (2006). Suppression of translation during in vitro maturation of pig oocytes despite enhanced formation of cap-binding protein complex eIF4F and 4E-BP1 hyperphosphorylation. Mol. Reprod. Dev..

[101] Tomek W., Melo Sterza F.A., Kubelka M., Wollenhaupt K., Torner H., Anger M., Kanitz W. (2002). Regulation of translation during in vitro maturation of bovine oocytes: The role of MAP kinase, eIF4E (cap binding protein) phosphorylation, and eIF4E-BP1. Biol. Reprod..

[102] Susor A., Jansova D., Cerna R., Danylevska A., Anger M., Toralova T., Malik R., Supolikova J., Cook M.S., Oh J.S. (2015). Temporal and spatial regulation of translation in the mammalian oocyte via the mTOR-eIF4F pathway. Nat. Commun..

[103] Jansova D., Koncicka M., Tetkova A., Cerna R., Malik R., del Llano E., Kubelka M., Susor A. (2017). Regulation of 4E-BP1 activity in the mammalian oocyte. Cell Cycle.

[104] Šušor A., Jelínková L., Karabínová P., Torner H., Tomek W., Kovářová H., Kubelka M. (2008). Regulation of cap-dependent translation initiation in the early stage porcine parthenotes. Mol. Reprod. Dev..

[105] Mayer S., Wrenzycki C., Tomek W. (2014). Inactivation of mTor arrests bovine oocytes in the metaphase-I stage, despite reversible inhibition of 4E-BP1 phosphorylation. Mol. Reprod. Dev..

[106] Severance A.L., Latham K.E. (2017). PLK1 regulates spindle association of phosphorylated eukaryotic translation initiation factor 4E binding protein, and spindle function in mouse oocytes. Am. J. Physiol. Cell Physiol..

[107] Lapasset L., Pradet-Balade B., Vergé V., Lozano J.C., Oulhen N., Cormier P., Peaucellier G. (2008). Cyclin B synthesis and rapamycin-sensitive regulation of protein synthesis during starfish oocyte meiotic divisions. Mol. Reprod. Dev..

[108] Ma X.M., Blenis J. (2009). Molecular mechanisms of mTOR-mediated translational control. Nat. Rev. Mol. Cell Biol..

[109] Fukunaga R., Hunter T. (1997). MNK1, a new MAP kinase-activated protein kinase, isolated by a novel expression screening method for identifying protein kinase substrates. EMBO J..

[110] Messina V., Di Sauro A., Pedrotti S., Adesso L., Latina A., Geremia R., Rossi P., Sette C. (2010). Differential contribution of the MTOR and MNK pathways to the regulation of mRNA translation in meiotic and postmeiotic mouse male germ cells. Biol. Reprod..

[111] Li Y., Yue P., Deng X., Ueda T., Fukunaga R., Khuri F.R., Sun S.-Y. (2010). Protein Phosphatase 2A Negatively Regulates Eukaryotic Initiation Factor 4E Phosphorylation and eIF4F Assembly through Direct Dephosphorylation of Mnk and eIF4E. Neoplasia.

[112] Shveygert M., Kaiser C., Bradrick S.S., Gromeier M. (2010). Regulation of Eukaryotic Initiation Factor 4E (eIF4E) Phosphorylation by Mitogen-Activated Protein Kinase Occurs through Modulation of Mnk1-eIF4G Interaction. Mol. Cell. Biol..

[113] Ellederová Z., Cais O., Šušor A., Uhlířová K., Kovářová H., Jelínková L., Tomek W., Kubelka M. (2008). ERK1/2 map kinase metabolic pathway is responsible for phosphorylation of translation initiation factor eIF4E during in vitro maturation of pig oocytes. Mol. Reprod. Dev..

[114] Brook M., Gray N.K. (2012). The role of mammalian poly(A)-binding proteins in co-ordinating mRNA turnover. Biochem. Soc. Trans..

[115] Ivshina M., Lasko P., Richter J.D. (2014). Cytoplasmic Polyadenylation Element Binding Proteins in Development, Health, and Disease. Annu. Rev. Cell Dev. Biol..

[116] Huarte J., Stutz A., O’Connell M.L., Gubler P., Belin D., Darrow A.L., Strickland S., Vassalli J.D. (1992). Transient translational silencing by reversible mRNA deadenylation. Cell.

[117] Nishimura Y., Kano K., Naito K. (2010). Porcine CPEB1 is involved in Cyclin B translation and meiotic resumption in porcine oocytes. Anim. Sci. J..

[118] Kim J.H., Richter J.D. (2006). Opposing Polymerase-Deadenylase Activities Regulate Cytoplasmic Polyadenylation. Mol. Cell.

[119] Fernández-Miranda G., Méndez R. (2012). The CPEB-family of proteins, translational control in senescence and cancer. Ageing Res. Rev..

[120] de Moor C.H., Richter J.D. (1997). The Mos pathway regulates cytoplasmic polyadenylation in Xenopus oocytes. Mol. Cell. Biol..

[121] Brunet S., Dumont J., Lee K.W., Kinoshita K., Hikal P., Gruss O.J., Maro B., Verlhac M.H. (2008). Meiotic regulation of TPX2 protein levels governs cell cycle progression in mouse oocytes. PLoS ONE.

[122] Yu C., Ji S.Y., Sha Q.Q., Dang Y., Zhou J.J., Zhang Y.L., Liu Y., Wang Z.W., Hu B., Sun Q.Y. (2016). BTG4 is a meiotic cell cycle-coupled maternal-zygotic-transition licensing factor in oocytes. Nat. Struct. Mol. Biol..

[123] Setoyama D., Yamashita M., Sagata N. (2007). Mechanism of degradation of CPEB during Xenopus oocyte maturation. Proc. Natl. Acad. Sci. USA.

[124] Keady B.T., Kuo P., Martínez S.E., Yuan L., Hake L.E. (2007). MAPK interacts with XGef and is required for CPEB activation during meiosis in Xenopus oocytes. J. Cell Sci..

[125] Komrskova P., Susor A., Malik R., Prochazkova B., Liskova L., Supolikova J., Hladky S., Kubelka M. (2014). Aurora kinase A is not involved in CPEB1 phosphorylation and cyclin B1 mRNA polyadenylation during meiotic maturation of porcine oocytes. PLoS ONE.

[126] Sha Q.-Q., Dai X.-X., Dang Y., Tang F., Liu J., Zhang Y.-L., Fan H.-Y. (2017). A MAPK cascade couples maternal mRNA translation and degradation to meiotic cell cycle progression in mouse oocytes. Development.

[127] Mulner-Lorillon O., Chassé H., Morales J., Bellé R., Cormier P. (2017). MAPK/ERK activity is required for the successful progression of mitosis in sea urchin embryos. Dev. Biol..

[128] Kracmarova J. (2017). Role of MAPK in Regulation of Cytoplasmic Polyadenylation during Meiotic Maturation of Mammalian Oocytes.

[129] Martins J.P.S., Liu X., Oke A., Arora R., Franciosi F., Viville S., Laird D.J., Fung J.C., Conti M. (2016). DAZL and CPEB1 regulate mRNA translation synergistically during oocyte maturation. J. Cell Sci..

[130] Eberhart C.G., Maines J.Z., Wasserman S.A. (1996). Meiotic cell cycle requirement for a fly homologue of human deleted in Azoospermia. Nature.

[131] Ruggiu M., Speed R., Taggart M., McKay S.J., Kilanowski F., Saunders P., Dorin J., Cooke H.J. (1997). The mouse Dazla gene encodes a cytoplasmic protein essential for gametogenesis. Nature.

[132] Eckerdt F., Pascreau G., Phistry M., Lewellyn A.L., DePaoli-Roach A.A., Maller J.L. (2009). Phosphorylation of TPX2 by Plx1 enhances activation of Aurora A. Cell Cycle.

[133] Helmke K.J., Heald R. (2014). TPX2 levels modulate meiotic spindle size and architecture in Xenopus egg extracts. J. Cell Biol..

[134] Bianchini A., Loiarro M., Bielli P., Busà R., Paronetto M.P., Loreni F., Geremia R., Sette C. (2008). Phosphorylation of eIF4E by MNKs supports protein synthesis, cell cycle progression and proliferation in prostate cancer cells. Carcinogenesis.

[135] Schlessinger J. (2000). Cell Signaling by Receptor Tyrosine Kinases A large group of genes in all eukaryotes encode for. October.

[136] Karar J., Maity A. (2011). PI3K/AKT/mTOR Pathway in Angiogenesis. Front. Mol. Neurosci..

[137] Mendoza M.C., Er E.E., Blenis J. (2011). The Ras-ERK and PI3K-mTOR pathways: Cross-talk and compensation. TRENDS Biochem. Sci..

[138] Lehr S., Kotzka J., Avci H., Sickmann A., Meyer H.E., Herkner A., Muller-Wieland D. (2004). Identification of major ERK-related phosphorylation sites in Gab1. Biochemistry.

[139] Rodriguez-Viciana P., Warne P.H., Dhand R., Vanhaesebroeck B., Gout I., Fry M.J., Waterfield M.D., Downward J. (1994). Phosphatidylinositol-3-OH kinase direct target of Ras. Nature.

[140] Dhillon A.S., Meikle S., Yazici Z., Eulitz M., Kolch W. (2002). Regulation of Raf-1 activation and signalling by dephosphorylation. EMBO J..

[141] Nakdimon I., Walser M., Fröhli E., Hajnal A. (2012). PTEN Negatively Regulates MAPK Signaling during Caenorhabditis elegans Vulval Development. PLoS Genet..

[142] McKay M.M., Morrison D.K. (2007). Integrating signals from RTKs to ERK/MAPK. Oncogene.

[143] Fissore R.A., He C.L., Vande Woude G.F. (1996). Potential role of mitogen-activated protein kinase during meiosis resumption in bovine oocytes. Biol. Reprod..

[144] Duncan F.E., Jasti S., Paulson A., Kelsh J.M., Fegley B., Gerton J.L. (2017). Age-associated dysregulation of protein metabolism in the mammalian oocyte. Aging Cell.

[145] Chao J.A., Yoon Y.J., Singer R.H. (2012). Imaging translation in single cells using fluorescent microscopy. Cold Spring Harb. Perspect. Biol..

[146] Romasko E.J., Amarnath D., Midic U., Latham K.E. (2013). Association of maternal mRNA and phosphorylated EIF4EBP1 variants with the spindle in mouse oocytes: Localized translational control supporting female meiosis in mammals. Genetics.

[147] Guertin D.A., Sabatini D.M. (2007). Defining the Role of mTOR in Cancer. Cancer Cell.

